# Regional disparities in access to assisted reproductive technology: assessment of patient satisfaction when employing modern technology to close the gap

**DOI:** 10.1007/s10815-020-02027-7

**Published:** 2021-01-14

**Authors:** Sasha Mikhael, Anna Gaidis, Larisa Gavrilova-Jordan

**Affiliations:** Department of Obstetrics & Gynecology, Section of Reproductive Endocrinology Infertility and Genetics, Augusta University, 1120 15th Street, BB-7518, Augusta, GA 30912 USA

**Keywords:** Telehealth, Assisted reproduction, Geographic disparities, Access to care

## Abstract

**Purpose:**

Geographic disparities for assisted reproductive technology (ART) continue to exist. Travel cost and time off work may create additional barriers for patients living remotely. Implementing telehealth can alleviate these barriers by reducing office visits. The aim of this study was to evaluate patient satisfaction with telehealth during ART.

**Methods:**

This was a cross-sectional survey and retrospective cohort study. Patients living remotely who underwent ART utilizing telehealth between 2015 and 2018 at a single institution were selected for the telehealth group. The non-telehealth control group included randomly selected patients who underwent IVF at the same institution between 2015 and 2018. Demographic variables and treatment outcomes were obtained for both groups. A patient satisfaction questionnaire was distributed to telehealth patients. Statistical analysis using *χ*^2^ test was performed to compare ART outcomes between both groups.

**Results:**

Ninety-seven control and 97 telehealth patients were included. For telehealth patients, the mean number of office visits and distance traveled was 2.9 (± 0.8 SD) and 143.1 miles (± 49.2 SD) respectively. 58.8% of patients completed the survey. 44/57 participants had an oocyte retrieval and 42/44 underwent embryo transfer. For those who completed the survey, the clinical pregnancy rate was 31/44 and the live birth rate was 25/44. There was no difference in treatment outcomes between telehealth compared to controls. 73% of patients were highly satisfied with telehealth.

**Conclusions:**

Telehealth can improve access to ART in underserved areas and results in high patient satisfaction. Reproductive health providers could consider telehealth as a safe and efficacious tool to ameliorate geographic disparities.

## Introduction

Many challenges exist in providing accessible and cost-effective reproductive care. According to the U.S. National Survey of Family Growth, only 38% of infertile women seek fertility services and only 60% of reproductive age women who require assisted reproductive technology (ART) for procreation are able to proceed [1]. Geographic barriers remain a key contributor to these statistics, with 18 million reproductive-aged women lacking any regional access to ART and another 7 million having access to only a single IVF clinic [2–4].

In the setting of ART, limited geographic access poses a unique challenge since multiple consultative appointments are often required along with frequent office visits for serial ultrasonography and blood draws during an IVF cycle. The majority of infertility clinics are located in densely populated cities with high median incomes as well as states where insurance coverage for fertility services is mandated [4, 5]. Meanwhile, there are states that have 0–1 IVF centers as reported by the CDC, leaving a considerable number of communities underserved [6].

When geographic disparities exist, modern technology can play a key role in bridging the gap. Telehealth is defined as the provision of health care services utilizing various technologic communication tools for prevention and/or treatment of disease [7]. Communication technologies include HIPAA compliant video conferences, phone calls, emails, text messages, faxes, and patient portals. According to the American Medical Association, the adoption of digital health by physicians has doubled between 2016 and 2019 increasing from 14 to 28% [11]. This tool has been validated in a number of specialties such as teleradiology and telepsychiatry, where 40% of radiologists and 28% of psychiatry practices offer telehealth services; however, it has not yet been widely adopted in reproductive health care [8, 9].

Though limited, the available evidence regarding use of telehealth in women’s reproductive care has demonstrated both safety and efficacy for services such as medical abortions and treatment of sexually transmitted infections [10–12]. One teleoncology study suggested telehealth may assist in overcoming geographic barriers and equalizing high-quality access to care when distance is the primary obstacle, particularly for women living 50 miles or greater from the closest gynecologist oncologist [13]. This is of particular importance since the increasing distance of residence from specialty centers was associated with reduced adherence to care with a subspecialist [14, 15]. In a systematic review analyzing telehealth interventions in obstetric and gynecologic health outcomes, 47 studies were included which encompassed all aspects of women’s health and its subspecialties. The review included low- and high-risk obstetrics, family planning, and general gynecology. The results highlighted improved health behaviors such as smoking cessation during pregnancy, compliance with contraception, and breastfeeding in addition to overcoming barriers for access to facility-based care [16]. Notably absent in this review was data concerning infertility treatments, highlighting the relative lack of research focused on telehealth as an intervention for improved access to ART.

Telehealth pertaining to assisted reproduction has scarcely been described in the literature. With little available evidence supporting its application for assisted reproduction, clinicians may feel reluctant to integrate telehealth in already complex treatment. The few studies that have evaluated this topic have suggested that telehealth can be strategically implemented in the setting of ART to reduce the burdens associated with travel and cost [17, 18]. One retrospective cohort study conducted in Spain sought to investigate the clinical advantages of using telehealth in the setting of ART. They demonstrated a significantly reduced wait time for evaluation and treatment with no difference in pregnancy or complication rates [17]. Another study described the successful use of 36 satellite programs for local monitoring in patients living remote from an IVF center, and though the study was limited by the exclusion of some common indications for IVF (e.g., male factor infertility), it demonstrated no differences in treatment outcomes between the satellite and central unit groups [18].

The application of telehealth for ART is in its infancy and specific data regarding utilization of telehealth by ART clinics is still lacking. Thus, despite the great potential for this tool, many important questions remain unanswered such as patient willingness to use the service, patient satisfaction, and treatment outcomes. The aim of this study was to assess the satisfaction of patients participating in a formal IVF telehealth program.

## Materials and methods

### ART telehealth program

The telehealth ART program was conducted utilizing the ARC® Telehealth System. Patient recruitment was based upon referrals following outreach to local gynecologists and general practitioners in rural areas. Prior to the initial consultation at our center, the patients’ local practitioners provided their completed history and work-up. Patients were offered telehealth for an initial consultation and/or follow-up appointments. IVF treatment protocols were individually selected based on patients’ clinical indications. Procedures such as saline infusion sonohysterogram, hysterosalpingograms, baseline ultrasound, and ovarian stimulation monitoring were performed by local gynecologists with results communicated to our office. Patients returned to our center for transvaginal oocyte retrieval and embryo transfer procedures.

### Study design

The study was a cross-sectional survey and retrospective cohort study performed at an academic fertility center at the Medical College of Georgia at Augusta University. The study was approved by Augusta University’s IRB.

### Patient selection

Patients living remotely, who underwent IVF using telehealth services between 2015 and 2018, identified via electronic medical records, were included in the telehealth group. Inclusion criteria consisted of patients utilizing telehealth services for ART treatment with fresh and frozen embryo transfers, with or without pre-implantation genetic testing (PGT). Patients with incomplete medical records or those who did not follow-up after the initial consultation were excluded since they did not pursue IVF at our facility. To help avoid sampling bias, only one cycle per patient was included. Demographic variables included the following: age, distance traveled to the clinic, and number of office visits. ART study variables included IVF procedures (oocyte retrieval, fresh and frozen embryo transfers), clinical pregnancy rate, and pregnancy outcome (live birth, spontaneous abortion). Women were grouped 18–24, 25–30, 31–34, 35–37, 38–39, 40–41, and over 41 years. Travel distance was categorized as follows: under 50, 50–100, 100–150, and greater than 200 miles.

The control group was obtained using a list of patients who underwent IVF from 2015 to 2018 with in-office visits. A random number generator was used to randomly select 97 patients. Patients who underwent an oocyte retrieval with fresh or frozen embryo transfer with or without PGT were included. No repeat IVF cycles for the same patient were included. Data obtained included clinical pregnancy rates and pregnancy outcomes (live birth, spontaneous abortion).

### Cross-sectional survey

A patient satisfaction survey was constructed for the telehealth group (Table 1), which included the same demographic variables as the chart review. The Qualtrics XM application was used to create the questionnaire, electronically distribute it, record responses anonymously, and analyze the data. Email and telephone reminders were provided to generate a higher response rate. The final analysis included data from subjects that completed the survey in its entirety.

**Table 1:** Patient satisfaction questionnaire distributed to patients identified in the electronic medical record to have undergone ART with telehealth

Question	Answers
1. Have you ever used telehealth for medical care at Reproductive Medicine and Infertility Associates (RMIA)?	Yes/no
2. What is your age?	a. 18–24
	b. 25–30
	c. 31–35
	d.36–37
	e.38–39
	f. 40–41
	g. > 41
3. How did you hear about RMIA?	a. Friend
	b. Physician referral
	c. Internet
	d. SART database
4. Did you undergo any infertility treatment at RMIA at Augusta University?	Yes/NO
5. In your infertility treatment course, how many times did you visit your provider at RMIA?	a. 0 times
	b. 1 time
	c. 2 times
	d. 3 times
	e. 4 times
	f. 5–9 times
	g. > 10 times
6. What is your travel distance?	a. < 50 miles
	b. 50–100 miles
	c. 100–150 miles
	d. 150–200 miles
	e. > 200 miles
7. How satisfied were you to have the opportunity to use telehealth?	a. Extremely satisfied
	b. Moderately satisfied
	c. Satisfied
	d. Neither satisfied nor dissatisfied
	e. Slightly dissatisfied
	f. Moderately dissatisfied
	g. Extremely dissatisfied
8. If dissatisfied, what specifically did you dislike about the telemedicine process?	
9. Would you recommend this service to others?	Yes/no
10 a. Were you aware that telemedicine was offered before choosing to obtain services at RMIA?	Yes/no
10 b. If you answered yes, did this influence your decision to come to RMIA	Yes/no
11. Did you have an egg retrieval?	Yes/no
12. Did you have an embryo transfer?	Yes/no
13. As a result of fertility treatments, did you get pregnant?	Yes/no
14. What was the result of your pregnancy?	a. Miscarriage
	b. Delivery
15. Would you come to RMIA for infertility treatment again?	Yes/no

### Statistical analysis

Treatment outcomes from the telehealth group and control group were compared using *χ*^2^ test with SPSS system version 26.

## Results

One hundred patients initially met inclusion criteria; however, 3 patients were excluded for not proceeding with ART. The median age for telehealth patients was 33 at the time of treatment compared to 34 in the control group. The mean number of office visits to RMIA for telehealth patients was 2.9 (± 0.8 SD), and the average travel distance was 143.1 miles (± 49.2 SD). Ninety-three percent (90/97) underwent transvaginal oocyte retrieval. The clinical pregnancy rate for all embryo transfers for the telehealth group was 60% (58/97) compared to 58% (56/97) in the control group outlined in Table 2 (*p* = 0.77). The overall live birth rate was 44% (43/97) versus 47% (46/97) in the control group (*p* = 0.67). The pregnancy loss rate was 10% (10/97) in both the telehealth and control groups. All women over 37 years of age or with recurrent pregnancy loss opted for PGT.

**Table 2:** Treatment outcomes comparing the telehealth ART group and controls who proceeded with local in-office visits throughout their IVF cycle

Treatment outcome	Telehealth, *n* (%)	Control, *n* (%)	*p* value
Clinical pregnancy	58/97 (60%)	56/97 (58%)	0.77
Live birth	43/97 (44%)	46/97 (47%)	0.67
Spontaneous abortion	10/97 (10.4%)	10/97 (10.4%)	1

The patient questionnaire was distributed electronically to the 97 patients in the telehealth group. Participants were provided with email and telephone reminders to complete the survey to improve the completion rate. There was an overall response rate of 72.2% (70/97), though 13/70 did not complete the survey and the questionnaire was completed in its entirety by 58.8% (57/97). Table 3 outlines the current age distribution of survey participants. For those who completed the survey, the mean number of office visits was 2.9 (± 0.8 SD), and the average travel distance was 143.1 miles (± 49.2 SD). Seventy-seven percent (44/57) of survey participants reported undergoing oocyte retrieval and an embryo transfer was performed in 95% (42/44) of these participants (Table 4). Two patients did not undergo embryo transfer due to the lack of viable embryos. The clinical pregnancy rate transfer was 70% (31/44) with a live birth rate of 57% (25/44).

**Table 3:** Patient demographics of survey participants including age and distance traveled to the office

Patient demographics		*n*
Age	< 35	35
	36–37	7
	38–39	6
	40+	9
Distance traveled	< 50	6
	50–100	8
	100–150	30
	150–200	9
	> 200	4

**Table 4:** ART treatment outcomes for survey participants who completed the survey in its entirety

Age (in years)	<35	36–37	38–39	40+	Total (*n*)
Oocyte retrieval	31	5	5	3	44
Embryo transfer	29	5	5	3	42
Clinical pregnancy	22 (71%)	4 (80%)	2(40%)	3 (100%)	31 (70%)
Live births	16 (52%)	4 (80%)	2 (40%)	3 (100%)	25 (57%)
Spontaneous abortion	6 (19%)	0	0	0	6 (14%)

**Fig. 1:**
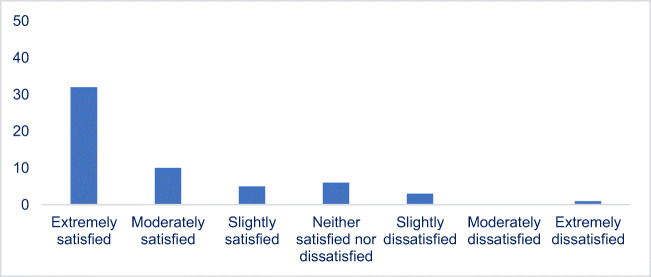
Patient satisfaction of survey participants regarding use of telehealth for ART services

Eighty-two percent (47/57) of survey responders reported being satisfied with telehealth services, as illustrated in Fig. 1. A total of 56% (32/57) were extremely satisfied, 17% (10/57) were moderately satisfied, 8% (5/57) were slightly satisfied, 10% (6/57) were neither satisfied nor dissatisfied, and 5% (3/57) were slightly dissatisfied. One patient was extremely dissatisfied; however, dissatisfied patients did not specify the reason for their dissatisfaction. Approximately 88% (50/57) of patients stated they would recommend telehealth for ART services to others. Seventy-nine percent (45/57) of patients were not initially aware of telehealth services at our facility. Of those 45 patients, 66.67% (38/57) reported that the availability of telehealth was a factor which positively influenced their decision to undergo IVF.

## Discussion

Geographic disparities due to the scarcity of IVF centers in rural areas can significantly impede access to ART for infertile patients. Due to the lack of IVF centers throughout central Georgia, study participants traveled an average of 143 miles to our office, approximately a 3-h drive one way. As a result, local monitoring was set up throughout central Georgia, with management of IVF cycles executed via telehealth. This significantly decreased frequency of travel during ovarian stimulation, reducing the number of office visits to an average of three, which included initial visit/review of IVF consent forms, oocyte retrieval, and embryo transfer. For frozen embryo transfers, appointments were conducted via telehealth, requiring only a single office visit for the embryo transfer. This infrastructure may help enable patients to proceed with IVF by alleviating the financial and physical burden of travel compounded with unpaid time off work [4, 19]. The results of this study demonstrated comparable treatment outcomes when using telehealth for ART. The overall clinical pregnancy rate for telehealth patients was 60% and the live birth rate of 44%, which was not statistically different from the control group. Of note, the low rates of spontaneous abortions in advanced maternal age women are reflected by clinical pregnancy rates associated with use of PGT. Based on these findings, telehealth may serve as a promising tool to reduce geographic barriers without compromising the quality of care.

To our knowledge, this is the first study to investigate patient satisfaction using telehealth for ART cycles in the USA. The questionnaire completed by patients revealed that 79% of patients were initially unaware of this service at our institution. Importantly, subsequent knowledge of telehealth services was a factor that positively influenced 67% of patients’ decision to proceed with necessary ART treatment. Patients demonstrated a high satisfaction rate with telehealth for their IVF cycles with 72% of patients stating they were moderately to extremely satisfied. A total of one survey participant stated that she was extremely dissatisfied and two participants stated they were slightly dissatisfied with the telehealth services. The survey contained a question to specify the reason for their dissatisfaction; however, this was not completed. Interestingly, survey participants reporting dissatisfaction were noted to have poor reproductive outcomes including lack of clinical pregnancy or pregnancy loss following embryo transfer, which could have contributed to their dissatisfaction. Overall, 88% of patients who underwent ART using telehealth stated they would recommend this service to others. Based on this data, outreach programs to inform patients, remote from an IVF center, of telehealth services may provide considerable benefit in improving access to high-quality care.

Though this was a descriptive cohort study to evaluate patient satisfaction of undergoing ART using telehealth, further prospective studies would be valuable in demonstrating the efficacy of this tool. Additional cost analysis comparing the use of telehealth to in-office care for ART cycles may also highlight other advantages of choosing telehealth for ART cycles.

Despite all of the undeniable benefits of telehealth in reproductive medicine, there are some logistical challenges worth noting. Many providers may find telehealth services burdensome when taking into account insurance coverage and reimbursement of services. Another commonly cited problem is licensure, particularly when caring for patients out of state [20]. Additionally, with limited scientific evidence to guide care using this technology, many physicians may resist adopting telehealth services, though integration of telehealth in reproductive medicine practices has been accelerated with the onset of the COVID-19 pandemic.

While the preliminary results of this study are promising, there are limitations to consider. This study included a small sample size making any real conclusions difficult to extrapolate. Additionally, despite a high initial response rate for the survey, responses were anonymous making it virtually impossible to target those who did not complete the questionnaire to further encourage its completion. Email and telephone reminders to all 97 subjects were made to help overcome this obstacle. It is also recognized that although telehealth can assist in overcoming geographic barriers, economic disparities can still significantly inhibit access to care. It is possible that cost of care and treatment outcomes may have impacted the patients’ perceived satisfaction of telehealth services, which was not taken into account. Additionally, since patients were self-selected, this could introduce a bias regarding the level of satisfaction.

In summary, telehealth can improve access to ART in underserved areas and results in high patient satisfaction. Reproductive health providers could consider telehealth as a safe and efficacious tool to ameliorate geographic disparities. Additional studies should be encouraged to narrow knowledge gaps and help guide evidence-based practices in “teleART.”

## Data Availability

Data is available upon request.
